# Gene Conversion in Human Genetic Disease

**DOI:** 10.3390/genes1030550

**Published:** 2010-12-22

**Authors:** Jian-Min Chen, Claude Férec, David N. Cooper

**Affiliations:** 1 Institut National de la Santé et de la Recherche Médicale (INSERM), U613, Brest, France; E-Mail: claude.ferec@univ-brest.fr; 2 Etablissement Français du Sang (EFS)-Bretagne, Brest, France; 3 Faculté de Médecine et des Sciences de la Santé, Université de Bretagne Occidentale (UBO), Brest, France; 4 Laboratoire de Génétique Moléculaire et d’Histocompatibilité, Centre Hospitalier Universitaire (CHU) de Brest, Hôpital Morvan, Brest, France; 5 Institute of Medical Genetics, School of Medicine, Cardiff University, Heath Park, Cardiff CF14 4XN, UK; E-Mail: cooperdn@Cardiff.ac.uk

**Keywords:** gene conversion mutation, homologous recombination, human inherited disease

## Abstract

Gene conversion is a specific type of homologous recombination that involves the unidirectional transfer of genetic material from a ‘donor’ sequence to a highly homologous ‘acceptor’. We have recently reviewed the molecular mechanisms underlying gene conversion, explored the key part that this process has played in fashioning extant human genes, and performed a meta-analysis of gene-conversion events known to have caused human genetic disease. Here we shall briefly summarize some of the latest developments in the study of pathogenic gene conversion events, including (i) the emerging idea of minimal efficient sequence homology (MESH) for homologous recombination, (ii) the local DNA sequence features that appear to predispose to gene conversion, (iii) a mechanistic comparison of gene conversion and transient hypermutability, and (iv) recently reported examples of pathogenic gene conversion events.

## 1. Introduction

**Figure 1:**
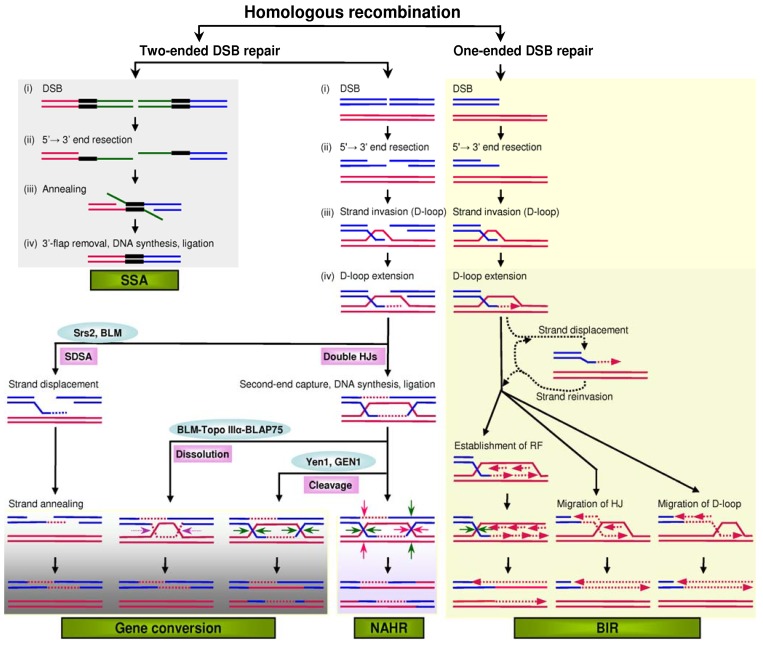
Mutational models of homologous recombination. In the models of gene conversion, NAHR (non-allelic homologous recombination) and BIR (break-induced replication), the invading strand invariably binds to a homologous sequence. In the model of SSA (single-strand annealing), the black bars indicate the direct repeats that flank a DSB (double-strand break). In the dissolution model of gene conversion, the two facing horizontal purple arrows indicate convergent branch migration. In the double HJs (Holliday junctions) cleavage model of gene conversion, the four horizontal green arrows indicate the orientation of resolution. In the double HJ cleavage model of NAHR, the dHJs can be cleaved as indicated by the green arrows or by the red arrows. In the model of BIR, the invading strand may undergo multiple rounds of displacement and annealing (indicated by dotted arrows) before a stable replication structure is established; this probably reflects repeated attempts to find the other end of the DSB. D-loop, displacement loop; RF, replication fork; SDSA, synthesis-dependent strand annealing. Reprinted from [1].

Gene conversion refers to the unidirectional transfer of genetic material from a ‘donor’ sequence to a highly homologous ‘acceptor’. It is one of four pathways of homologous recombination, the other three being non-allelic homologous recombination (NAHR), break-induced replication (BIR) and single-strand annealing (SSA) (Figure 1). All pathways share a similar initiating event: The double-strand break (DSB) generated within one of the duplicated (or repeated) sequences undergoes extensive 5'-end resection to form 3' single-stranded DNA tails. Gene conversion, NAHR and SSA all serve to repair DSBs with two ends, whereas BIR repairs DSBs with only one end. Gene conversion and NAHR may be considered to represent alternative outcomes of a common two-ended DSB repair process (for detailed description, see [1]).

In a recent review article, we assessed the current thinking in relation to the molecular mechanisms underlying gene conversion, surveyed the impact of gene conversion on human genome evolution, and performed a meta-analysis of pathogenic gene conversion events [2]. In this article, we shall briefly summarize some of the latest advances in the study of pathogenic gene conversion events.

## 2. The Emerging Idea of Minimal Efficient Sequence Homology for Homologous Recombination

Homologous recombination is one of the major mechanisms for the repair of DSBs (the other is non-homologous end joining [1]). As the term implies, homologous recombination is mediated through sequences which exhibit considerable similarity that presumably serves to stabilize chromosomal mispairing. In this regard, it is pertinent to note that the rate of gene conversion is directly proportional to the length of the uninterrupted sequence tract in the putatively converted region: in mouse cells, the minimal efficient processing segment (MEPS) for efficient meiotic homologous recombination is >200 bp [3,4] while in humans, it is estimated to be in the range of 337–456 bp [5]. 

How extensive does the global sequence similarity need to be for efficient homologous recombination to occur between two interacting sequences? Our analysis of 44 interlocus pathogenic gene conversion events revealed that the similarity between the interacting sequences is almost invariably >92% [2]. This finding has recently received strong support from the results of a study that approached this issue from an evolutionary perspective [6]. Both the human and chimpanzee growth hormone gene (*GH1* in humans and *GHN* in chimpanzee) promoters are highly polymorphic, and all 14 human *GH1* promoter SNPs and five of the nine chimpanzee *GHN* promoter SNPs could potentially have resulted from interlocus gene conversion (*i.e*., the minor allele occurs in at least one of the *cis*-linked paralogous genes); by contrast, no polymorphism was evident in the macaque *GH1* gene promoter. Remarkably, the mean degree of pair-wise similarity between the *GH1* promoter and its paralogs in macaque is 92.0%, significantly lower than in either chimpanzee (93.5%) or human (94.0%). Thus, it appears that if the degree of similarity between related gene sequences falls below a certain threshold (perhaps around 92%), then gene conversion may be significantly reduced or even abolished, with attendant consequences for the genetic variability manifested by the sequences in question [6].

By analogy to the concept of MEPS, we have proposed that efficient homologous recombination (including gene conversion) may also require a minimal efficient sequence homology (MESH; approximately 92%) between the interacting sequences [7]. Further supporting evidence for this idea has come from the accurate estimation of copy number variation and multicopy gene number in 159 human genomes; signatures consistent with gene conversion were virtually exclusive to high-identity duplications (>95% sequence similarity) and tended to occur in association with tandem duplications (≤1 Mbp) [8].

In summary, whereas the concept of MEPS defines a local sequence property of homologous recombination, MESH defines a global sequence property of homologous recombination. 

## 3. Local Sequence Features Predisposing to Gene Conversion

A variety of DNA sequences, including direct repeats, inverted repeats (sometimes incorrectly termed palindromes), minisatellite repeats, the χ recombination hotspot, and alternating purine–pyrimidine tracts with Z-DNA-forming potential, have frequently been noted in association with gene conversion in human genes (see references in [9]). In addition, the convergence of biochemical, genetic, and genomic studies in the context of gross genomic deletions, inversions, duplications, and translocations has suggested that the ability of a given DNA sequence to adopt a non-B DNA conformation (e.g., slipped structures, triplexes and tetraplexes), rather than the DNA sequence *per se* (in the orthodox right-handed Watson-Crick B-form), could induce chromosomal DSBs (reviewed in [10]). However, no methodical statistically based analysis had been performed to formalize these observations until recently, when a series of well-characterized human gene conversion mutations were employed as a test system. The advantage of this novel approach lay in the fact that the extents of the maximal converted tracts (MaxCTs) and minimal converted tracts (MinCTs) associated with such pathological events could usually be fairly accurately determined and annotated [9]. *In silico* analysis of the DNA sequence tracts involved in 27 nonoverlapping pathogenic gene conversion events in 19 different genes yielded several novel findings [9]. First, gene conversion events tend to occur preferentially within (C + G)- and CpG-rich regions. Second, sequences with the potential to form non-B DNA structures occur disproportionately within MaxCTs and/or short flanking regions. Third, MaxCTs are enriched (*P* < 0.01) in a truncated version of the χ element (a TGGTGG motif), immunoglobulin heavy chain class switch repeats, translin target sites and several novel motifs including (or overlapping) the classical meiotic recombination hotspot, CCTCCCCT. Finally, gene conversions tend to occur in genomic regions that have the potential to fold into stable hairpin conformations [9]. These findings therefore provide support for the concept that recombination-inducing motifs, in association with alternative DNA conformations, can promote recombination in the human genome.

**Figure 2:**
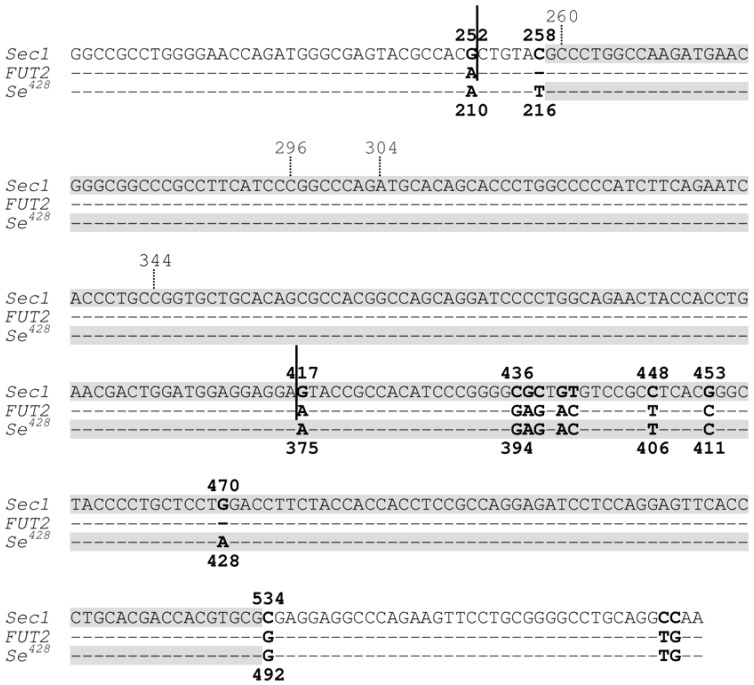
Partial sequence alignment of the *Sec1* gene, the *FUT2* gene and the *Se^428^* mutant allele of the *FUT2* gene. Dashes indicate identity with *Sec1* DNA sequence. Nucleotides are numbered in accordance with previous publications (e.g., [11]) for easy comparison; numbers above the aligned sequences refer to *Sec1* sequence whereas those below the aligned sequences refer to both *FUT2* and *Se^428^* sequences. As compared with the wild-type *FUT2* gene, the *Se^428^* mutant allele contains the 428G > A nonsense mutation and the 216C > T polymorphism. Sequence between the two vertical continued bars indicates the crossover region of the non-allelic homologous recombination-derived *Se^fus^* allele, in which the 3'-part of the *FUT2* gene was fused to the 5'-part of the *Sec1* gene. Shaded sequences indicated the maximal converted tract (MaxCT) of the gene conversion-derived *Sec1*-*Se^428^*-*Sec1* allele (*Sec1* is the acceptor gene whilst *Se^428^* is the donor gene). The overlapping sequence tract between the MaxCT of the *Sec1*-*Se^428^*-*Sec1* allele and the crossover region of the *Se^fus^* allele spans positions 259 to 416 in the context of the *Sec1* sequence. Reprinted from [13].

The importance of non-B DNA conformations in predisposing genomic rearrangements is perhaps best exemplified by a rather unique case of gene conversion. Soejima and colleagues reported a *Sec1*-*FUT2*-*Sec1* hybrid allele that apparently resulted from a gene conversion event [11]. As pointed out by these authors, this allele is more appropriately termed *Sec1*-*Se^428^*-*Sec1* because it is the *Se^428^* mutant allele of the *FUT2* gene that acts as the donor sequence (Figure 2). Interestingly, the 5' half of the MaxCT of this interlocus gene conversion event overlaps with the crossover region of the previously reported *Se^fus^* mutant allele (Figure 2). The *Se^fus^* allele was generated by NAHR via a process through which the 3'-part of the *FUT2* gene was fused to the 5'-part of the *Sec1* gene [12]. Based on our current understanding of mutational mechanisms, we reasoned that the initiating DSBs leading to the *Sec1*-*Se^428^*-*Sec1* and *Se^fus^* alleles might have occurred within the aforementioned overlapping sequence tract.

**Figure 3:**
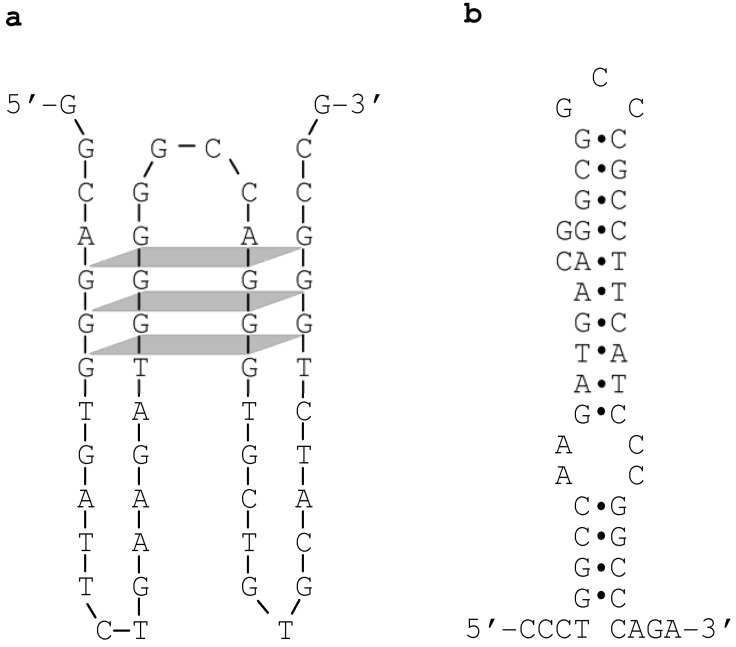
Non-B conformations identified within the overlapping sequence tract between the maximal converted tract (MaxCT) of the *Sec1-Se^428^-Sec1* allele and the crossover region of the *Se^fus^* allele (refer to Figure 2). (**a**) A tetraplex structure formed by four GGG repeats. The sequence illustrated corresponds to the reverse complement of *Sec1* spanning positions 296 to 344 (see Figure 2); (**b**) A hairpin structure formed by a pair of imperfect inverted repeats. The sequence illustrated corresponds to *Sec1* spanning positions 260 to 304 (see Figure 2). Reprinted from [13].

We further reasoned that, were this to be the case, the overlapping sequence tract might be capable of adopting non-B conformation(s) [13]. Indeed, upon inspection, four GGG repeats within the overlapping sequence tract were identified that would appear to have the potential to fold into a tetraplex structure (Figure 3a). In addition, by means of a previously established method for predicting the ‘local’ secondary structure of nucleotide sequences [14], we identified a hairpin structure that can potentially be formed by a specific pair of imperfect inverted repeats (Figure 3b). We speculate that these non-B DNA structures may have acted either individually or synergistically to promote the formation of DSBs, which in turn could have initiated the process of homologous recombination [13].

## 4. Gene Conversion *vs* Transient Hypermutability: A Mechanistic Comparison

Data from a wide variety of organisms (including viruses, prokaryotes and yeast, as well as cell lines and tissues from higher eukaryotes) have clearly demonstrated that the number of instances of multiple mutation is significantly higher than would be predicted simply from the mutation frequency and a random distribution of mutations [15]. For example, some of the multiple somatic *HPRT* mutations detected in a human epithelial cell line were closely spaced, with 4/12 mutation pairs being separated by only 6 bp on average, a much higher proportion than would be expected by chance alone [16]. The most robust data came from studies employing the Big Blue^®^ transgenic mouse system [17,18]; complete sequencing of the 1.4 kb *lacI* transgene in thousands of mouse mutants from normal tissues and spontaneous tumors demonstrated that the distribution of the spacing between component mutations in doublets (two spatially separated mutations identified in *cis*) was highly non-random, with half the doublets being separated by <120 bp [18]. 

**Figure 4:**
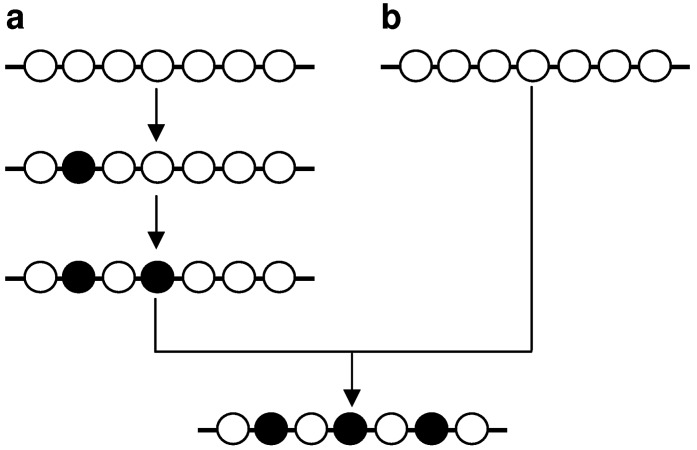
Two concepts for generating multiple mutations. Multiple mutations can accumulate during multiple cell cycles (**a**) or can be generated in the same cell cycle and in rapid succession (**b**). Adapted from [22].

‘Multiple mutations’ can in principle be the observable net result of the sequential accumulation of single mutations independently generated during multiple cell replications (Figure 4a). However, known examples of such mutations [19,20] exhibit an essentially random inter-component spacing distribution, as would be expected for mutations of independent origin [15,21]. Consequently, the multiple mutations that exhibit non-random proximal spacing in higher eukaryotes [16,18]—termed ‘closely spaced multiple mutations’ (CSMMs; [22])—are most compatible with a model in which they are generated simultaneously or quasi-simultaneously in the same cell cycle (Figure 4b). Multiple synchronous mutations have been postulated to arise via transient hypermutability resulting from (i) the deregulated expression of, or conformational change in, either a replicative DNA polymerase or another protein involved in the maintenance of replication fidelity, (ii) the disruption of the balance of the nucleotide pool, or (iii) the recruitment of error-prone DNA polymerases in DNA replication or repair [15,23,24]. 

Recently, we have extended the concept of transient hypermutability from somatic cells to the germline, using human inherited disease-causing multiple mutations as a model system. Employing stringent criteria for data inclusion, we retrospectively identified 151 potential examples of pathogenic CSMMs [22]. Taken at face value, these examples possessed at least three features which were consistent with those noted with the Big Blue^®^ transgenic mouse system [17,18]. First, a large fraction of the multiple mutations were closely spaced. Second, some single nucleotide substitutions (SNS) were found to coexist with other types of mutation. Finally, a small fraction of the collected multiple mutations comprised three or more distinct components. In particular, eight multiple mutations comprised three or more components within a sequence tract of <100 bp. The majority of these mutations may reasonably be assumed to have occurred as simultaneous or quasi-simultaneous events, thereby providing the first evidence to support the contention that the human germline can also experience transient hypermutability [22].

We then sought to procure evidence to support the postulate that the closely spaced double mutations causing human inherited disease arose predominantly through transient hypermutability. For reasons of simplicity, we focused our attention upon the 102 disease-causing double mutations that comprise exclusively SNS mutations. Transient hypermutability has been postulated to result from three different mechanisms (see before), all of which imply new DNA synthesis. One mutational mechanism which does not involve new DNA synthesis is methylation-mediated deamination of 5-methylcytosine, which gives rise to C > T transitions (or G > A transitions on the complementary strand). Since 5-methylcytosine in the human genome is almost exclusively confined to the CpG dinucleotide, this mechanism accounts for the CpG dinucleotide being a mutation hotspot. We therefore surmised that the proportion of CpG substitution, manifested by the component mutations from a given set of multiple mutations, could be used as a crude indicator of the relative likelihood of transient hypermutability: the lower the proportion of CpG substitution, the higher the likelihood that the multiple mutations would have arisen via transient hypermutability [22].

**Figure 5:**
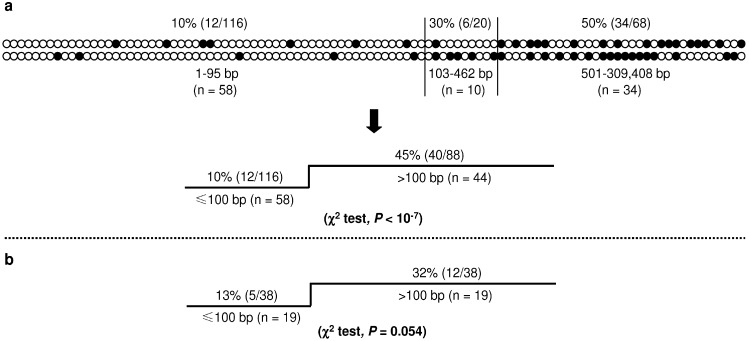
CpG substitutions in double single nucleotide substitution (SNS) mutations. (**a**) Top panel illustrates the distribution of CpG substitutions in 102 pathogenic double SNS mutations. Each pair of vertical circles indicates a double SNS mutation. The upper and lower circles indicate the first and second components of a given double mutation, respectively. Solid circles indicate CpG substitutions. The lower panel compares the proportion of CpG substitutions in the group of ≤100 bp with that in the group of >100 bp; (**b**) Proportion of CpG substitutions in the group of ≤100 bp with that in the group of >100 bp; data were derived from a re-analysis of all double SNS mutations obtained from the Big Blue mice [17,18]. Reprinted from [22].

The 102 double SNS mutations could be roughly divided into three groups on the basis of the relative proportion of CpG substitution (top panel, Figure 5a). The first group comprised 58 events with an inter-component distance of 1–95 bp, and had the lowest proportion of CpG substitution (10%). The second group comprised 10 events with an inter-component distance of 103–462 bp, with an intermediate CpG substitution rate of 30.0%. The third group, comprising the remaining 34 events with an inter-component distance of 501–309,408 bp, exhibited the highest CpG substitution rate (50%). This distribution pattern coincided with two observations made in the Big Blue transgenic mice: first, nearly all the observed doublets were separated by an intervening sequence of <500 bp; second, it was those doublets with an inter-component distance of 1–100 bp that occurred more frequently than would be expected for independent mutations [18]. Adopting a fairly conservative strategy, we used the cutoff value of ≤100 bp to define CSMMs in the human context, manifesting a CpG substitution rate of 10%, significantly lower than that the 45% which characterized the remaining 44 double SNS events of >100 bp (lower panel, Figure 5a). Employing the same standard, we also revisited the double SNS mutations reported by Buettner *et al.* [17] and Hill *et al.* [18] and derived a *P* value of marginal significance: CpG substitution rates were 13% for the 19 events of ≤100 bp and 32% for the 19 events of >100 bp (Figure 5b).

The aforementioned findings strongly suggest that the two groups of double SNS mutations (*i.e*., ≤100 bp and >100 bp) arose via qualitatively quite different mutational mechanisms. This postulate was then given further support from the analysis of the highly informative homocoordinate mutations (multiple mutations in the same gene involving the same mutation type but occurring at different sites in *cis* [18]). Of the 102 double SNS mutations causing inherited disease, 17 were found to be homocoordinate mutations. Again, qualitative differences were apparent between the two groups of homocoordinate mutations. Only one of the six homocoordinate events in the ≤100 bp group involved a CpG substitution. By contrast, 10 of the 11 homocoordinate events in the >100 bp group were characterized by CpG substitutions [22].

Taking these data together, we proposed that CSMMs comprising at least one pair of mutations separated by ≤100 bp may constitute signatures of transient hypermutability in human genes [22]. Here it should however be emphasized that gene conversion events (with the exception of those that induce only a single nucleotide change) constitute in effect an important type of multiple mutation. They are also thought to be generated simultaneously or quasi-simultaneously in the same cell cycle; moreover, their MaxCTs are usually short, rarely exceeding 1 kb [2]. Mechanistically, there is a qualitative difference between these gene conversion events and multiple mutations originating via transient hypermutability. Whereas gene conversion constitutes a template-switching event through which a highly homologous template is faithfully copied by a normal replicative DNA polymerase, transient hypermutability-mediated multiple mutations are due to misincorporation of bases during DNA replication or repair.

## 5. New Examples of Pathogenic Gene Conversion Events

We initially collated 44 interlocus gene-conversion events (involving a total of 17 genes) that are known to have given rise to human inherited disease (see Table 1 in [2]). In the process of collating examples of multiple mutations that could have arisen through transient hypermutability [22], we identified eight further gene conversion mutations, all of which were included in our subsequent *in silico* analysis of the local sequence features that might predispose to gene conversion [9]. Here it is important to emphasize that all of these gene conversion events were collected in accordance with fairly stringent selection criteria. For example, all reported gene-conversion events comprising only a single nucleotide substitution were omitted from the analysis, since the possibility that these changes may have originated by simple point mutation can never be excluded. In addition, all gene conversion events that were not fully characterized at the nucleotide sequence level were also excluded [2]. Our collation therefore represents only a fraction of the actual number of pathogenic gene conversion events already described in one form or another in the literature. Hence, these collated data allow only a very conservative estimate to be made of the likely relative frequency of gene conversion as a cause of human inherited disease.

In a just published report, Boria and colleagues investigated the possible occurrence of pseudogene-mediated gene conversion in Diamond-Blackfan anemia (DBA) [25]. Mutations in nine ribosomal protein (*RP*) genes have so far been reported in ~50% of DBA patients. They aligned sequences of the most frequently mutated *RP* genes (*i.e*., *RPS19*, *RPL5* and *RPL11*) with their respective pseudogene sequences and looked at 5 bp on each side of the mutation; coincidence was found in six mutations (Table 1). Evidently, they “could not exclude that the same changes arose independently in the gene and in the pseudogene” [25]. Here we would like to add that (i) both micro-deletions occurred within short direct repeats (three A and two AGAC, respectively) such that they are explicable by the classical model of replication slippage, a subclass of the recently coined “microhomology-mediated replication-dependent recombination (MMRDR)” mechanism [1], and (ii) three (*i.e*., c.403G > A, c.166C > T, and c.535C > T) of the four SNS mutations are CpG substitutions. By contrast, the c.191T > C mutation did not occur within a known mutational hotspot and thus may have a higher probability of being generated by gene conversion.

**Table 1:** Six ribosomal protein (RP) mutations found to coincide with pseudogene sequences.

Gene (Chromosomal Localization)	Mutation	Wild-Type Sequence	Pseudogene Sequences	Pseudogene (Chromosomal Localization)
*RPS19* (19q13.2)	c.384_385delAA	GGACA **AA**GAGAT	GGACA **--**GAGAT	*RPS19P2* (1p13.2)
	c.403G > A	GAATC **G**CCGGA	GAATC **A**CCGGA	*RPS19P2* (1p13.2)
	c.191T > C	GCACC **T**GTACC	GCACC **C**GTACC	*RPS19P4* (5q11.2)
	c.166C > T	ACACG **C**GAGCT	ACACG **T**GAGCT	*RPS19P7* (10q11.21)
*RPL5* (1p22.1)	c.535C > T	CCAAA **C**GATTC	CCAAA **T**GATTC	*RPL5P34* (22q13.2)
*RPL11* (1p36.1-p35)	c.94_97delAGAC	GAGAC **AGAC**TGAG	GAGAC **----**TGACG	*RPL11P5* (12q24.31)

* From [25]

In certain cases, gene conversion is nevertheless more plausible than simple point mutation as an explanation for the observed single nucleotide changes. For example, Moradkhani *et al.* identified 14 different human hemoglobin (Hb) variants resulting from identical mutations on either one of the two human α-globin paralogous genes (*HBA1* and *HBA2*) [26]. Interallelic gene conversion was regarded as the most plausible mechanism to account for “the same mutation being ‘transferred’ into different genomic contexts”. In support of this postulate is the fact that 13 out of the 14 Hb variants were located within exons 1 and 2; these two exons (but not exon 3) have been previously shown to be involved in gene conversion events [26]. Another example is provided by the identification of high frequency sequence exchange events between *PMS2* and its pseudogene, *PMS2CL*, in which gene conversion has certainly played a key role [27]. 

Recently, Gardner *et al.* [28] reported a pathogenic gene conversion mutation that satisfies our previously established stringent selection criteria [2]. In a family with X-linked cone and cone-rod dystrophies, a missense mutation (c. 529T > C [p. W177R]) in exon 3 of both the long-wavelength-sensitive and medium-wavelength-sensitive cone opsin genes (*OPN1LW* and *OPN1MW*) was found to segregate with the disease. As opined by Gardner *et al.*, the spontaneous occurrence of this point mutation in both genes is most unlikely. Much more likely was that the mutation first originated in one gene and was then transferred to the other by a gene conversion event. Indeed, the mutation in the *OPN1LW* gene was found to be embedded within a block of *OPN1MW* sequence [28] (a gene conversion mutation involving *OPN1LW* and *OPN1MW* was previously reported to cause blue cone monochromacy [29]). This new gene conversion event and the eight events described in Chuzhanova *et al.* [9] are summarized in Table 2. Additionally, a putative double gene conversion event causing spinal muscular atrophy has also been reported [30]. 

**Table 2:** Recently collated examples of interlocus gene conversion events.

Disease/Phenotype	Donor Gene	Acceptor Gene	Chromosomal Localization	Mutation	Ref.
Congenital adrenal hyperplasia	*CYP21A1P*	*CYP21A2*	6p21.3	Intron 2 conversion	[31]
Increased *CYP3A7* expression in adult liver and intestine	*CYP3A4*	*CYP3A7*	7q21-q22.1	Promoter conversion	[32]
Novel St glycophorin	*GYPE*	*GYPA*	4q28-q31	GPA-E-A hybrid gene	[33]
Microcytosis	*HBA2*	*HBA1*	16p13.3	*α121* patchwork	[34]
Agammaglobulinemia	*IGLL3*	*IGLL1*	22q11.23	Conversion of exon 2	[35]
*Sec1*–*FUT2*–*Sec1* hybrid allele	*FUT2*	*Sec1*	19q13.3	Conversion involving exonic sequence	[11]
Atypical hemolytic uremic syndrome	*CR1L*	*CD46*	1q32	D151N + Y155D	[36]
Pachyonychia congenita type 2	*KRT17P3*	*KRT17*	17q21.2	452G > A and 457T > C	[37]
X-linked cone and cone-rod dystrophies	*OPN1MW*	*OPN1LW*	Xq28	c. 529T > C [p. W177R]	[28]

* Collated after the publication of [2]; the first eight entries have been previously described in [9].

## 6. Conclusions

Further gene conversion mutations causing human inherited disease will continue to be identified in the future and these should contribute to our emerging understanding of this important mutational mechanism. Indeed, the identification and analysis of such naturally occurring gene conversion events will serve as an invaluable source for refining the general characteristics of gene conversion in particular and homologous recombination in general. Although, to date, most relevant research has focused on the study of germline mutations, we speculate that somatic mosaicism resulting from interallelic gene conversion, which has until now largely escaped our attention, could turn out to be a potentially new and important modifier of human inherited disease [2]. Finally, it is pertinent to mention that an artificial form of gene conversion, brought about by means of homing endonucleases, holds great promise for targeted gene therapy in patients with monogenic diseases (reviewed in [38]).
